# Lymph nodes—The neglected battlefield in tuberculosis

**DOI:** 10.1371/journal.ppat.1008632

**Published:** 2020-08-13

**Authors:** Sharie Keanne C. Ganchua, Alexander G. White, Edwin C. Klein, JoAnne L. Flynn

**Affiliations:** 1 Department of Microbiology and Molecular Genetics, University of Pittsburgh School of Medicine, Pittsburgh, Pennsylvania, United States of America; 2 Division of Laboratory Animal Resources, University of Pittsburgh, Pittsburgh, Pennsylvania, United States of America; 3 Center for Vaccine Research, University of Pittsburgh School of Medicine, Pittsburgh, Pennsylvania, United States of America; University of Georgia, UNITED STATES

## Abstract

Lymph nodes, particularly thoracic lymph nodes, are among the most common sites of extrapulmonary tuberculosis (TB). However, *Mycobacterium tuberculosis* (Mtb) infection in these organs is understudied. Aside from being sites of initiation of the adaptive immune system, lymph nodes also serve as niches of Mtb growth and persistence. Mtb infection results in granuloma formation that disrupts and—if it becomes large enough—replaces the normal architecture of the lymph node that is vital to its function. In preclinical models, successful TB vaccines appear to prevent spread of Mtb from the lungs to the lymph nodes. Reactivation of latent TB can start in the lymph nodes resulting in dissemination of the bacteria to the lungs and other organs. Involvement of the lymph nodes may improve Bacille Calmette-Guerin (BCG) vaccine efficacy. Lastly, drug penetration to the lymph nodes is poor compared to blood, lung tissue, and lung granulomas. Future studies on evaluating the efficacy of vaccines and anti-TB drug treatments should include consideration of the effects on thoracic lymph nodes and not just the lungs.

## Introduction

Tuberculosis (TB) is an ancient disease that has plagued humans for thousands of years [1]. It has claimed millions of lives, killing 1.45 million people in 2018 alone, making it the leading cause of death by a single infectious agent. It is caused by bacteria, *Mycobacterium tuberculosis* (Mtb), which are spread in aerosolized droplets expelled from symptomatic individuals, i.e., those with active TB [2]. Recent estimates suggest that approximately one-quarter of the world’s human population is currently infected with this microbe without symptomatic and microbiological evidence of disease, which is clinically defined as latent TB [3]. Even though TB most commonly manifests as a pulmonary disease, extrapulmonary TB also occurs. In humans, Mtb infection usually results in a Ghon complex—a tuberculous lung lesion accompanied by a granuloma in a thoracic lymph node [4, 5]. Infected lymph nodes are considered to be extrapulmonary, even if they are within the thoracic cavity, and are the most common sites of extrapulmonary Mtb infection [6, 7]. Lymph nodes are niches for Mtb growth and persistence [8]. Early autopsy studies in humans found live Mtb in lymph nodes without signs of TB disease anywhere else in the body [9–11]. Even lymph nodes that appeared normal through gross inspection by a trained pathologist could harbor live Mtb [9]. In cattle, lymph nodes are the most common site of *M*. *bovis* infection [12]. In a small study of 15 cattle with evidence of bovine TB in lymph nodes, only 1 had identifiable pulmonary infection [13]. However, some authors as cited by Neill and colleagues [12] believe that a more comprehensive inspection of the bovine lungs should be performed since TB lesions can be small. It is widely accepted that, in bovine TB, lymph nodes get infected first while pulmonary lesions develop later during the infection [12, 14]. In our experience working with nonhuman primates (NHPs), lymph nodes are almost always infected with Mtb along with the lungs [8]. Occasionally, we find lymph nodes with no apparent granuloma also harboring live Mtb bacilli. Given these observations, it is understandable that Behr and Waters proposed TB as a lymphatic disease rather than strictly a pulmonary disease [15].

Reviews of human TB lymphadenitis (TBLN) focusing on epidemiology, clinical manifestations, pathology, diagnosis, and treatment have been published [16–20]. Here, we aim to review the pathogenesis of Mtb infection in lymph nodes, drawing on studies from animal models and humans.

### From the air to the lymph nodes

Infection begins when Mtb enters the airways in inhaled droplet nuclei expelled from individuals with active TB disease. Poulsen published 2 extensive studies in the 1950s detailing the early events in Mtb infection in 517 tuberculin skin test (TST) converters in the Faroe Islands [21, 22]. At the time that Poulsen conducted his study, this group of islands just north of the United Kingdom had a population of 30,000 living in isolated villages. A version of TST was done routinely on all inhabitants, and detailed medical histories were recorded. He determined that the incubation period—i.e., the time from Mtb exposure to the first clinical sign of infection (e.g., fever, erythema nodosum [reddish nodules of inflammation on the skin], TST conversion, X-ray showing hilar adenopathy or lung abnormalities)—is around 40 days. The first sign of infection was almost always onset of fever [21, 23]. The changes seen in chest radiographs were observed early, often coincident with the initial fever, and these changes consisted mainly of enlarged and dense hilar shadows. The hila is composed of pulmonary arteries and veins, major bronchi, and lymph nodes. The common causes of enlarged hila are (1) lymphadenopathy and tumors, (2) arterial or venous hypertension, and (3) increase in pulmonary blood flow [24]. Often, these hilar changes remained for 1–2 years before receding. Pulmonary infiltrates were not as common, present only in a little more than one-third of children and less than one-third of adults [22], although the radiograph technology at the time was unlikely to be sufficient to detect small initial lung lesions. Of the 517 TST converters, 333 (64%) showed hilar lymphadenitis, which occurred more in children than in adults (78% of children versus 56% of adults). However, after prolonged observation, only approximately 10% of the TST converters developed clinically defined active TB, indicating that the early events involving lymph nodes and lungs occur in a large percentage of people following infection, even though only a fraction of these will go on to develop active disease.

**Fig 1:**
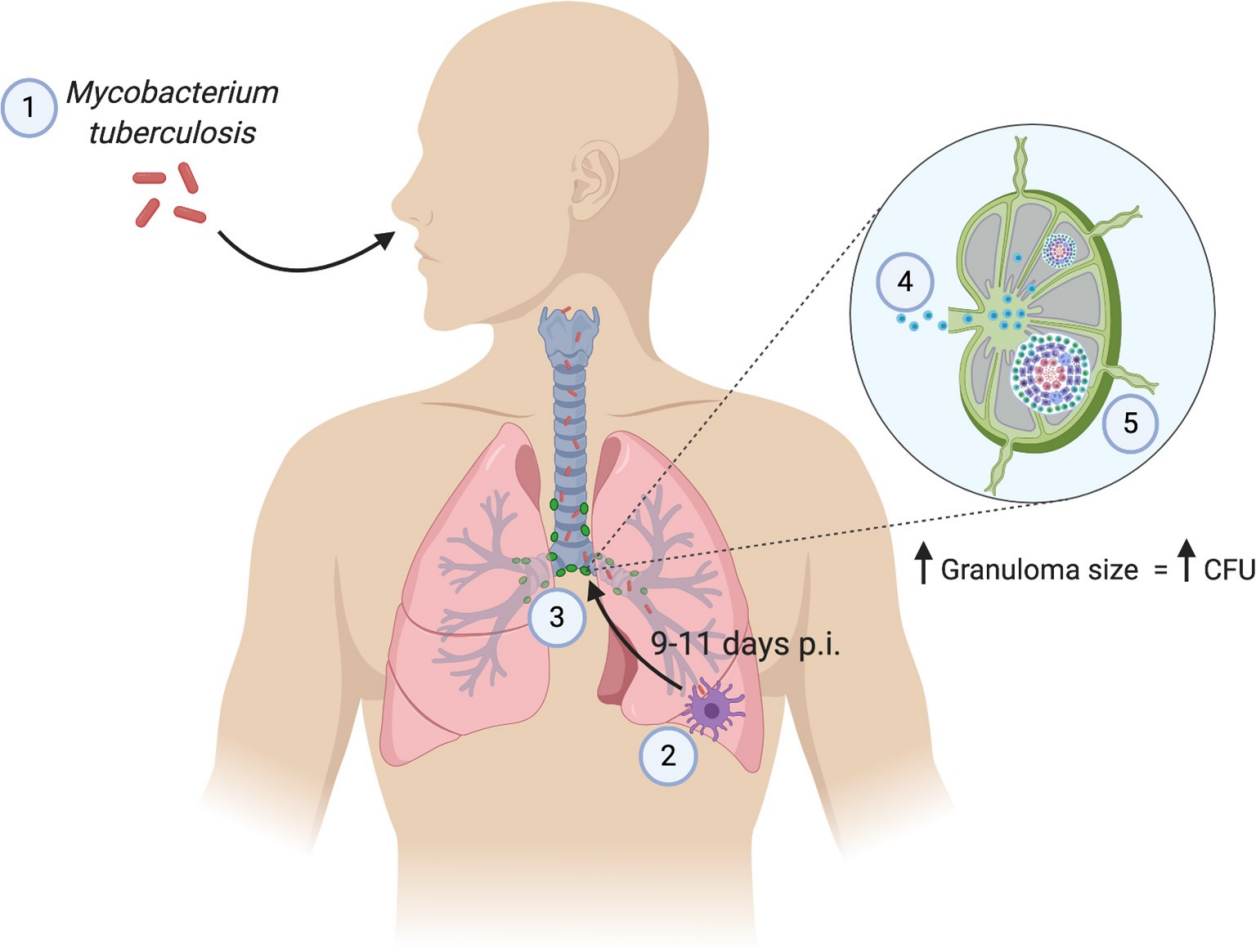
Mtb travels to the thoracic lymph nodes from the lungs. (1) Infection begins when a person inhales aerosolized droplets containing Mtb. (2) Mtb travels into the lungs and is taken up by phagocytic cells. (3) Mtb is then transported to a thoracic lymph node on the same side of the body. Steps 2 and 3 take 9–11 days in mice. (4) Mtb-containing phagocytic cells present antigen to naïve lymphocytes and generate an immune response. Activated lymphocytes travel back to the lungs to contain Mtb infection. (5) Live Mtb, either shuttled to the lymph nodes by phagocytic cells or carried by lymph fluid, begins to multiply and cause a granuloma to form. Mtb burden increases as the granuloma size increases. Lymph nodes are generally not able to eliminate the infection. CFU, colony forming units; Mtb, *Mycobacterium tuberculosis*; p.i., post infection.

The involvement of lymph nodes during the first month of Mtb infection is well established in mouse models of TB. After aerosol infection, Mtb is phagocytosed by alveolar macrophages, myeloid dendritic cells (DC) and neutrophils in the lungs [25]. While other respiratory viral and bacterial pathogens induce DC migration to the lymph nodes to activate the adaptive immune system by 1–3 days post infection [26–28], this important process is delayed in Mtb infection. Several studies have shown that Mtb-infected DCs do not migrate to the lymph node and prime T cells until 9–11 days post infection (Fig 1) [29–31]. This delay in the dissemination of Mtb bacteria to the lymph nodes is thought to play a role in the increased susceptibility of C3H/HeJ mice to Mtb compared to C57BL/6 mice [29]. Wolf and colleagues also showed that the migration of DCs was transient, slowing down after peaking at 21 days post infection, an interesting observation given the chronic nature of TB. Not only are DC migratory functions dysregulated, but DCs and interstitial macrophages that transport Mtb to the lymph nodes are relatively poor at stimulating T-cell responses to Mtb antigens [30].

Lymph nodes are a major component of early Mtb infection in guinea pigs aerosolly infected with 20 colony forming units (CFU) [32–34]. One of the earliest (5–15 days post infection) observations in the lungs is inflammation of pulmonary lymphatic vessels [33]. Marked thoracic lymph node enlargement could be seen around 20 days post infection progressing to severe lymphadenopathy at 30 days post infection [32, 34]. Lymph node involvement has also been noted in rabbit models [35–37].

NHPs, particularly cynomolgus macaques, are excellent experimental TB models since they present with the full spectrum of human clinical TB (latent to active TB) when challenged with a low dose (≤25 CFU) and form granulomas identical to those formed in humans [38–46]. Although rhesus macaques are more susceptible and generally develop active TB, their TB pathology also recapitulates that of humans [47–49]. Macaques also have multiple thoracic lymph nodes that presumably drain different sections of the lungs, just like in humans, as opposed to rodents having only a few lung-draining lymph nodes. NHPs provide an excellent model for studying the involvement of thoracic lymph nodes in Mtb infection.

By performing serial positron emission tomography coupled with computed tomography (PET-CT) scans, we can track Mtb infection in the lungs and lymph nodes of NHPs over the course of infection [47, 50–55]. We used a radiolabeled glucose analog, ^18^F-fluorodeoxyglucose (FDG), as our PET probe which is taken up and retained by metabolically active cells. FDG activity is a surrogate marker for inflammation in lung granulomas and lymph nodes [47, 50–55]. In contrast to lung granulomas, lymph nodes can be difficult to detect by PET-CT unless they are enlarged or FDG avid (metabolically active) [47, 53]. In macaques, one or more thoracic lymph nodes start to become FDG avid 2–4 weeks post infection, as do the lung granulomas [8, 52, 53]. These FDG-avid thoracic lymph nodes could reflect immune cell activation or proliferation in response to priming, as well as an active site of Mtb infection. Combining PET-CT data with quantitative bacterial burden assessments in NHP, we reported that when thoracic lymph nodes were “hot” (SUVR, or maximum standard uptake ratio normalized to muscle ≥ 5), 96.3% contained culturable Mtb bacilli; however, only 50% of “warm” thoracic lymph nodes (SUVR ≥ 2.3 but < 5) had live Mtb. Interestingly, 40 of 240 lymph nodes that were not detectable by PET (SUVR < 2.3) also had culturable Mtb [8, 52]. In a previous study examining the early events of Mtb infection in cynomolgus macaques, granulomas assessed grossly were observed to form first in the thoracic lymph nodes before being detectable in the lungs [39]. Macaques euthanized at 3 weeks post infection had bilaterally enlarged hilar lymph nodes; however, no gross nor microscopic granuloma were seen in the thoracic lymph nodes or lungs of these animals. Macaques euthanized at 4 weeks post infection had bilaterally enlarged hilar lymph nodes, but only 1 of 2 macaques had a small granuloma grossly visible in the enlarged hilar lymph node. At this time point, multiple thoracic lymph nodes showed evidence of infection ranging from early (i.e., aggregates of epithelioid macrophages, sometimes with multinucleated giant cells) to more advanced (i.e., multifocal and coalescing areas of inflammation with central necrosis). One macaque had 1 granuloma in the lungs that was seen microscopically. By 5 to 6 weeks post infection, macaques exhibited greater pathology in both thoracic lymph nodes and lungs. Caseous granulomas were visible in both lungs and thoracic lymph nodes by gross assessment. T-cell responses in thoracic lymph nodes to mycobacterial proteins (culture filtrate protein [CFP]) can be detected by ELISpot at 3–4 weeks post infection, and this generally preceded responses in the blood and lungs. These observations suggest that pathology may progress from the thoracic lymph nodes to the lungs, and this coincides with the adaptive immune response being activated in the lymph nodes first before trafficking to the lungs [39]. However, it should be noted that this early study was done without the benefit of PET-CT imaging, and it is very possible that early small granulomas in lungs were missed during necropsy.

Once Mtb enters the airways and the lungs, only a fraction (9/98, 9.2%) gets transported to one or more thoracic lymph nodes and successfully infects them. By using DNA barcoded Mtb strains that allow discrimination of individual bacteria, Martin and colleagues [56] tracked infection dynamics in lungs and thoracic lymph nodes of cynomolgus macaques. While the majority of lung granulomas were formed by a single bacterium (only 1 barcode was found per granuloma), most lymph nodes were infected with multiple bacteria (≥2 barcodes). However, only a fraction (9/98, 9.2%) of the barcodes found in lung granulomas were also identified in culturable bacteria from lymph nodes. This suggests that after replicating in the lungs, either not all of Mtb that seeded granulomas were also able to disseminate to the lymph nodes and successfully replicate there, or they were transported to the lymph nodes but did not establish a productive infection.

### Bacterial dynamics in the lymph nodes

To the authors’ knowledge, there is currently limited existing comprehensive analysis of Mtb bacterial dynamics in lymph nodes of small animal models. The reports indicate that Mtb gets shuttled into the lymph nodes mostly by DCs and interstitial macrophages around 9–11 days post infection [29–31], but since most of these studies were focused on the early events of T-cell priming, mice were euthanized at around day 28, and the fate of Mtb in these lymph nodes during long-term infection is unknown. In these T-cell priming studies, mice were infected at varying doses (<15 to 555 CFU), and the peak CFU was detected around days 14–21 post infection at approximately 10^5^ to 10^6^ numbers [25, 29–31, 57]. The degree and timing of peak CFU was correlated to the magnitude of the inoculation dose. A longer-term study on the effect of Bacille Calmette-Guerin (BCG) on Mtb burden in various tissues found that Mtb inoculated into the ear reaches peak CFU in ear-draining lymph nodes around 28 days post infection in unvaccinated C57BL/6 mice and remains relatively constant at 10^3^ CFU until 120 days post infection [58]. Another study that followed Mtb infection in resistant (B10.MBR) and susceptible (B10.SM) strains of mice showed a rapid increase of Mtb to approximately 10^4^ CFU in the thoracic lymph node 3 weeks post infection, which increased to 10^4^–10^5^ CFU by 10 weeks post infection [59]. In guinea pigs, Mtb can be cultured from lymph nodes in very low numbers (approximately 100 CFU) as early as 5 days post infection, reaching a peak at 20 days post infection (10^6^ CFU) before decreasing and stabilizing by 60 days at 10^5^ CFU [60]. These studies suggest that in mice and guinea pigs, once Mtb enters the lymph node, the host is unable to eliminate it, making the lymph node a bacterial reservoir.

Studying Mtb bacterial dynamics in human lymph nodes is extremely difficult since the timing of Mtb infection is usually unknown and thoracic lymph node biopsies are invasive. However, NHP models provide an opportunity to dissect the dynamics of lung and lymph node infections since infection timing and dose are known, and necropsies can be performed at various time points post infection. The 2 species of macaques most commonly used to model TB respond differently to Mtb infection. Following low dose infection, about half the cynomolgus macaques develop active TB disease, and the other half develop latent Mtb infection (defined as no clinical signs of disease and negative Mtb cultures from bronchoalveolar lavage (BAL) and gastric aspirate over 6 months) [40]. Rhesus macaques, on the other hand, are more susceptible to Mtb infection, always developing active TB disease when infected with a fully virulent strain of Mtb [47]. One difference between the 2 species is how their lymph nodes respond to Mtb infection. Rhesus macaque lymph nodes present with more extensive pathology and greater increases in size such that the lymph nodes can impinge on the airways, sometimes leading to lobe collapse. A recent publication from our group showed that live Mtb burden reaches its peak at 4–6 weeks post infection in both cynomolgus and rhesus macaques [8]. In cynomolgus macaques, this Mtb burden is reduced 100-fold by 11–14 weeks post infection and remains constant until 16–29 weeks. In cynomolgus macaques that had latent Mtb infection (34–54 weeks post infection), one or a few lymph nodes had Mtb bacilli, and the bacterial numbers were significantly fewer compared to earlier time points post infection. In contrast, although rhesus macaque lymph nodes had approximately 10-fold-lower Mtb burden at 4 weeks post infection compared to cynomolgus macaques, this level of bacteria is maintained until 16–28 weeks post infection. We determined the chromosomal equivalents (CEQs; Mtb genomes quantified by quantitative PCR [qPCR]) in each lymph node as an approximation of the total number of bacteria (counting both live and dead Mtb) and found equivalent numbers in both macaque species at nearly all time points post infection. The ratio of live Mtb (CFU) to CEQ can be used to determine the killing capacity of each lymph node [61]. For both macaque species, there was minimal Mtb killing in the lymph nodes at 4–6 weeks post infection; however, cynomolgus macaque lymph nodes were able to kill at least a portion of Mtb at later time points post infection. The killing capacity of rhesus macaque lymph nodes never improved even at 16–28 weeks post infection. Thus, Mtb grew to the same level in the lymph nodes of both macaque species; however, rhesus macaque lymph nodes were not successful at killing Mtb, contributing to the more severe disease in the lymph nodes of these animals. We also found Mtb DNA in peripheral lymph nodes (axillary and inguinal) which do not drain the lungs. Since most of these lymph nodes were sterile and did not have granulomas apparent by histopathology, it seems that they have a high capacity for killing Mtb. However, trafficking of dead Mtb or Mtb genomes to these lymph nodes is also possible. In general, lymph nodes are poor killers of Mtb compared to lung granulomas [8, 61].

### Immune response of lymph nodes to Mtb

Once Mtb has reached the lymph nodes and an adaptive immune response is generated, the lymph node needs to contain or kill the growing number of Mtb bacteria inside it. Otherwise, the lymph node can be destroyed by necrosis. The primary mechanism to achieve bacterial killing is likely through the production of cytokines, chemokines, cytolytic and other effector molecules by cells in the lymph nodes [62, 63]. Although our study showed limited to no killing in the majority of thoracic lymph nodes in cynomolgus and rhesus macaques, we still found sterile lymph nodes with granulomas, albeit small in number (16 out of 200 [8%] lymph nodes with granulomas by microscopic histopathology were sterile). Thus, some lymph nodes are successful in killing Mtb; however, this is a rare occurrence. Comparing successful and unsuccessful immune response of lymph nodes may provide clues to immune control of TB.

Human studies investigating the immune response in Mtb-infected lymph nodes compared biopsied cervical lymph nodes of patients with TBLN with either healthy controls, patients with only pulmonary TB, and patients with other lymph node disease (e.g., cancer, non–TB-specific reactive lymphadenitis) [64–67]. All studies obtained transcription profiles, but only one study examined protein levels. Moreover, only one study obtained data on bacterial burden in the lymph node samples from the TBLN patients. With these caveats in mind, we can only view these findings as the lymph node’s response to Mtb infection without knowing whether that response was successful in killing the bacteria. In general, unstimulated cervical lymph nodes from patients with TBLN exhibited up-regulated transcripts related to viral defense, inflammatory response, toll-like receptor (TLR) signaling, tumor necrosis factor (TNF) signaling, and Th1-associated pathways compared to lymph nodes from healthy controls or patients with pulmonary TB only, TB meningitis, or lymph node cancer [64, 65]. Down-regulation of Th2 pathways was also observed [65]. When stimulated with Mtb-specific antigens, CFU+ lymph nodes had higher interleukin-10 (IL-10), T helper type 1 (Th1), T helper type 17 (Th17), and granulocyte-macrophage colony-stimulating factor (GMCSF) protein levels compared to CFU-lymph nodes from TBLN patients. No difference in levels of Th2 cytokines (IL-4, IL-5, and IL-13), IL-1β, or IL-18 was observed [66]. In contrast to other studies, Rahman and colleagues [67] showed that lymph nodes from children with TBLN had lower interferon alpha (IFNα), TNF, and IL-17 expression compared to non–TB-specific reactive lymph nodes and healthy tonsil controls. However, forkhead box protein 3 (Foxp3), transforming growth factor beta (TGFβ), and IL-13 mRNA were increased in lymph nodes from TBLN children. No changes in IL-4 or IL-10 were detected. Different experimental measures (mRNA versus protein), technique (microarray versus qPCR), samples and controls (TBLN patients versus variety of samples used as controls), and patients (adults versus children) could all contribute to variability in findings. The aggregate data support that lymph nodes respond to Mtb infection in a variety of ways, but whether these responses promote growth or killing of Mtb is unknown.

We examined Th1 (IFN-γ, TNF, IL-2), Th17 (IL-17), and IL-10 cytokine expression from T cells, B cells, and CD11b+ cells in thoracic lymph nodes of 24 cynomolgus macaques in response to Mtb antigen (6 kDa early secretory antigenic target [ESAT-6] and CFP-10) stimulation [8]. Uninfected lymph nodes (no granuloma by gross inspection or histopathology and no live Mtb) had higher proportions of CD3+ T cells than lymph nodes with granulomas. This is likely due to the destruction of lymph node architecture by granulomas. When compared to lymph nodes with live Mtb (CFU+), lymph nodes that were able to clear Mtb (with granulomas but CFU negative) had a significantly higher proportion of CD11b+ cells producing IL-10. On the other hand, CFU positive lymph nodes had a higher proportion of CD4+ T cells producing TNF. A significant negative correlation was found between IL-10-producing CD11b+ cells and bacterial burden, while a weak but significant positive correlation was found between CD4+ T cells producing TNF and bacterial burden. These data suggest that bacterial clearance is associated with CD11b+ macrophages producing IL-10 while TNF-producing CD4 T cells is associated with Mtb replication. The presence of IL-10 can be beneficial to lymph nodes as a balance of pro-inflammatory and anti-inflammatory signals and is associated with bacterial clearance in lung granulomas [68]. Neutralizing IL-10 in cynomolgus macaques also resulted in higher Mtb burden in lymph nodes at 4 weeks post infection [69].

### Mtb remodels lymph node structure

**Fig 2:**
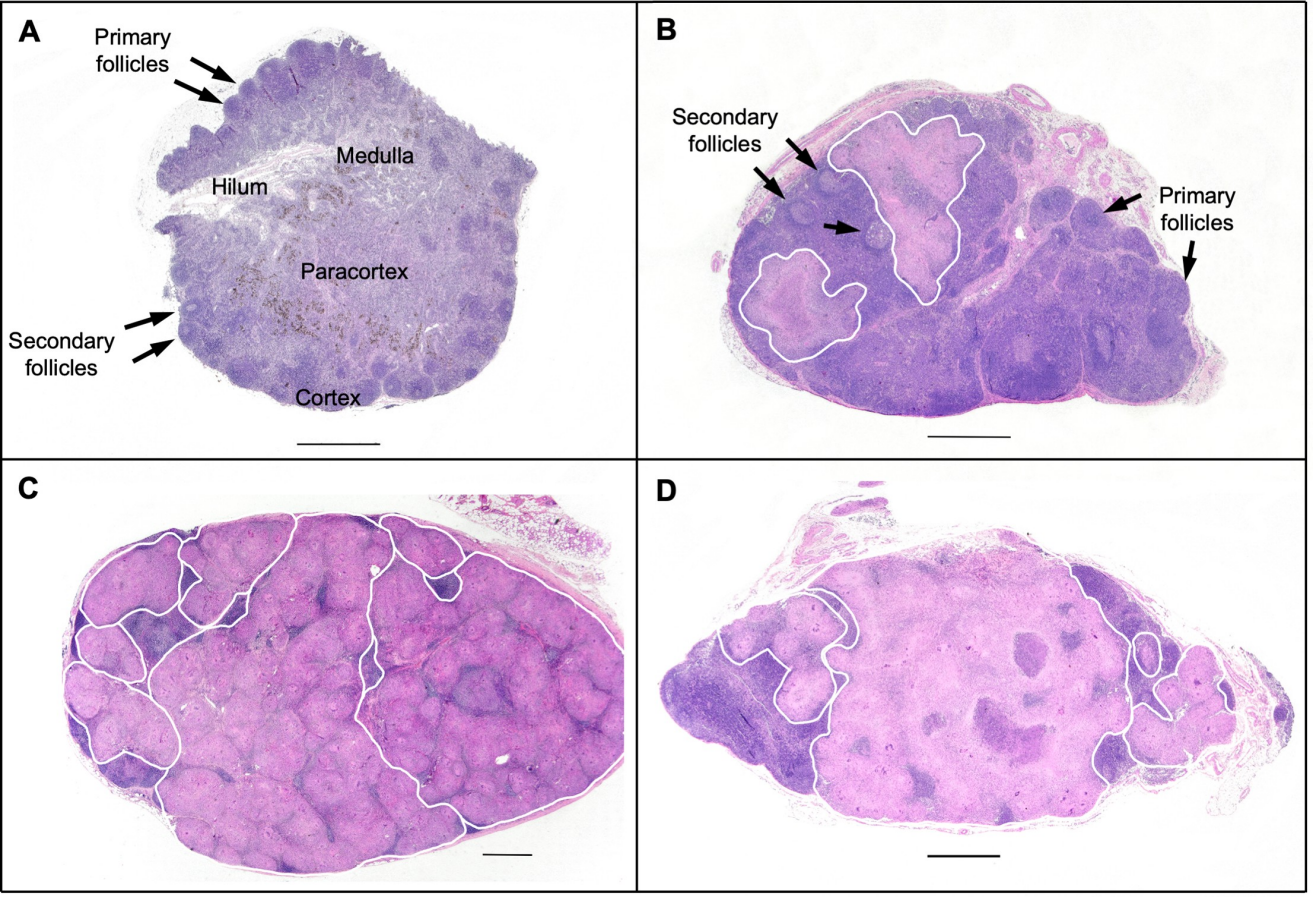
Mtb infection results in granuloma formation that disrupts the normal lymph node architecture. (A) Lymph node showing normal architecture without granuloma formation. (B) Lymph node with partially effacing granulomas. (C) Total nodal effacement by multiple coalescing non-necrotizing granulomas. (D) Near total nodal effacement by multiple coalescing caseous granulomas. Granulomas are outlined with a white line. Measuring bar = 1 mm. Mtb, *Mycobacterium tuberculosis*.

Lymph nodes are organs whose function is tightly linked to their architecture. Different types of cells have predetermined spaces they call “home” (e.g., B cells in follicles, T cells in paracortex, macrophages distributed throughout the cortex, subcapsular sinus, and medullary region) (Fig 2A). Antigen-presenting cells interact with T and B cells in set locations, and this facilitates initiation of the adaptive immune response [70–74]. Mtb infection of lymph nodes result in formation of granulomas, either in separate foci or coalescing, that destroy the lymph node’s architecture (Fig 2B–2D). We provided evidence that in lymph nodes where ≥50% of the area is occupied by a granuloma or coalescing granulomas, Mtb burden is higher compared to lymph nodes with granuloma(s) occupying <50%. This is true whether we examined live Mtb burden (CFU) or total Mtb burden (live and dead Mtb; CEQ). Minimal killing (CFU/CEQ) was observed irrespective of extent of granuloma involvement. Even a small granuloma composed of clusters of macrophages can push T cells out of their normal spatial arrangement, impinge on germinal centers, and disrupt the normal vasculature in these organs [8]. Similar disruption of lymph node architecture has been shown in humans with TBLN [67]. Granulomas that form in lymph nodes are structurally distinct from granulomas that form in the lungs. We showed that even though lymph node granulomas form in the T-cell and B cell regions of the lymph node, they lack B cell–rich tertiary lymphoid structures that form in the periphery of a lung granuloma. Distinct lymphocyte cuff regions found in lung granulomas are also negligible in lymph node granulomas. These observations suggest that the structural and compositional differences between lymph node and lung granulomas could be related to the poor Mtb killing potential of lymph nodes [8].

We compared the cytokine response (Th1, Th17, IL-10) and proliferation (Ki67) of CD4+ and CD8+ T cells to Mtb-specific antigens in lymph nodes without granulomas, with <50% granuloma involvement, and with >50% involvement. There was no difference in any of the cytokines or Ki67 measured among all groups, suggesting that the size of the granuloma inside a lymph node does not affect the overall function of the lymph node T cells [8]. Using immunohistochemistry, lymph nodes of human patients with TBLN also displayed extensive remodeling and enrichment of macrophages and DCs, with relatively stable T-cell proportions, while the number of B cells was reduced compared to patients with non–TB-specific reactive lymph nodes [67].

### Mtb disseminates ipsilaterally from lungs to lymph nodes

**Fig 3:**
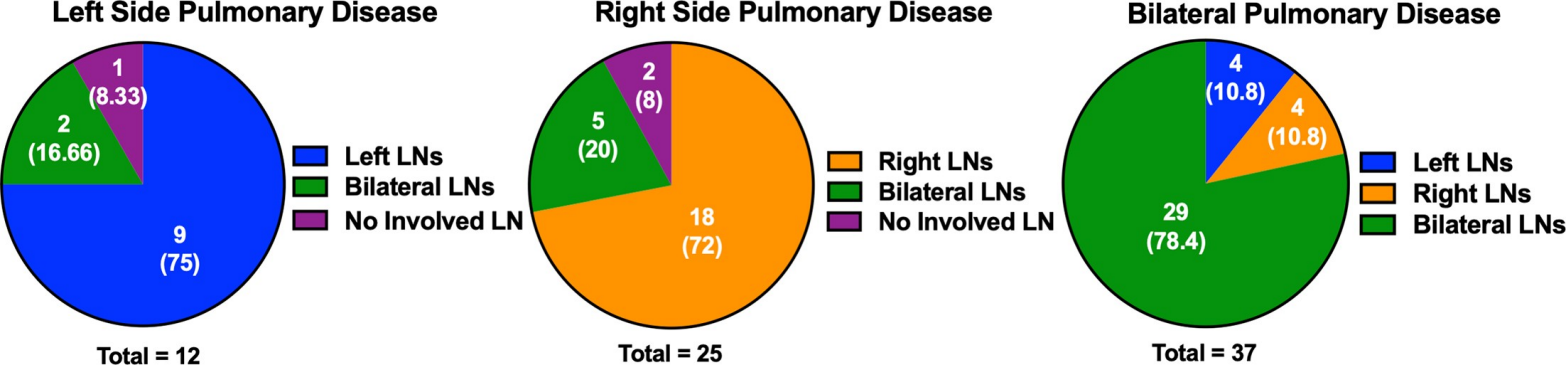
Mtb is spread ipsilaterally from the lungs to the thoracic lymph nodes in macaques. Cynomolgus macaques infected with low dose Mtb necropsied from 10 to 55 weeks post infection were assessed for lung disease by gross pathology during necropsy. Lymph node involvement was determined by quantitative culture for Mtb. The pie charts show the lymph node counts. The proportion is in parenthesis. *N* = 74 monkeys. LN, lymph node; Mtb, *Mycobacterium tuberculosis*.

To determine the pattern of Mtb dissemination from lungs to lymph nodes, we examined whether macaques that formed lung granulomas in the right lung lobes had live Mtb in thoracic lymph nodes on the right side (ipsilateral), left side (contralateral), or both sides (bilateral) of the airways. We assessed the presence of granulomas in each lung lobe and bacterial burden in each lymph node obtained during necropsy from 74 cynomolgus macaques 10–55 weeks post infection. The majority of macaques that formed granulomas on one side of the lungs (approximately 75%) had CFU+ lymph nodes on the same side of the airways (Fig 3). Most macaques that had granulomas on both sides of the lungs also had CFU+ lymph nodes on both sides of the airways (Fig 3). Only a small proportion (approximately 20%) of macaques that had granulomas in only one side of the lungs had bilateral lymph involvement. This suggests that Mtb primarily travels ipsilaterally (same side) from the lungs to the lymph nodes. Variability in draining of the lungs by lymph nodes, airway involvement, and lymph node to lymph node spread could explain the bilateral lymph node involvement in macaques with unilateral lung granulomas.

These NHP data are consistent with human data from an early study on 283 autopsies of children [4] and is consistent with the anatomy of the lymphatic system draining the lungs. In general, the right lung drains to the lymph nodes on the right side, and the left lung drains to the lymph nodes on the left, with the exception of the left lower lobe, which might cross over to the right via the lower tracheobronchial lymph nodes [75, 76].

### Lymph nodes are sites of TB reactivation

**Fig 4:**
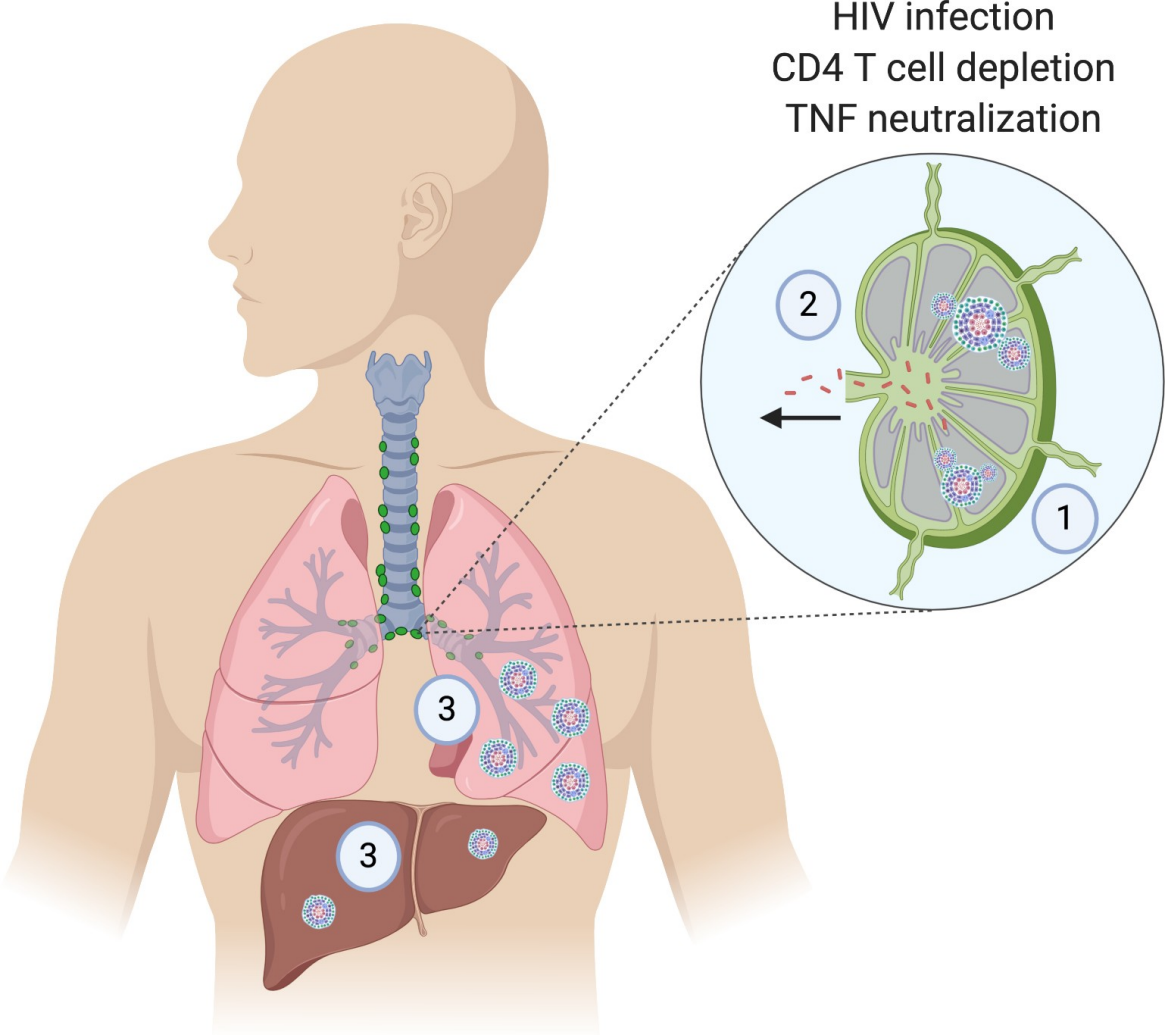
Latent TB reactivation can start in the lymph nodes. Mtb resides undetected in lymph nodes during latent Mtb infection or as a result of inadequate drug concentration in lymph nodes during treatment. (1) After latent TB reactivation is induced by HIV infection, CD4 T-cell depletion, or TNF neutralization, non-necrotizing granulomas form adjacent to established and often mineralized granulomas in the lymph nodes. (2) Mtb exits lymph nodes through unknown mechanisms, probably carried by lymph into the subclavian vein and then spreading hematogenously or when the lymph node structure breaks down and delivers bacilli to the airways. (3) Mtb travels to and forms new granulomas in the lungs and other organs (extrapulmonary TB). Mtb, *Mycobacterium tuberculosis*; TB, tuberculosis; TNF, tumor necrosis factor.

Since true latency and reactivation models are either nonexistent or not well studied in small animal models, especially in relation to lymph nodes, we did not include them in this section. Based on human and macaque studies, lymph nodes can play a major role in reactivation of latent TB caused by immunosuppression. In NHPs, we define reactivation TB as a positive culture in BAL and/or gastric aspirate, increase in erythrocyte sedimentation rate, signs of disease such as coughing or weight loss, or the formation of a new granuloma by PET-CT after latent Mtb infection was established [51, 77–80]. In CD4 T-cell–depleted cynomolgus macaques, lower CD4+ T-cell levels in hilar lymph nodes was associated with reactivation [80]. In TNF-neutralized macaques, early signs of reactivation (i.e., non-necrotizing granuloma formation adjacent to established and often mineralized granulomas) were observed microscopically in the lymph nodes [78]. Latently Mtb-infected macaques with a high risk of reactivating after TNF neutralization had a smaller proportion of sterile thoracic lymph nodes, highly metabolically active (by PET-CT) lymph nodes, and increased live Mtb burden in lymph nodes compared to low-risk animals [51]. In a separate study [79], DNA barcoded Mtb bacteria, which allows for the discrimination of individual bacteria, was used to track Mtb dissemination during reactivation of latent TB (latent TB defined in our laboratory as animals with no clinical signs or culturable BAL or gastric aspirate and normal erythrocyte sedimentation rate up to 6 months post-Mtb infection [38, 39, 78]) in cynomolgus macaques induced by simian immunodeficiency virus (SIV) co-infection. New lung granulomas that arose during reactivation were assessed for DNA barcodes and compared to the DNA barcoded bacilli found in old granulomas (those present prior to SIV infection) or in thoracic lymph nodes. Almost 50% of the DNA barcodes in new granulomas matched DNA barcodes from bacteria only found in lymph nodes and not in the old granulomas. Moreover, Mtb recovered from extrapulmonary sites (e.g., liver and spleen) had the same barcodes as Mtb from the lymph nodes. This suggests that Mtb dissemination during reactivation can originate from the lymph nodes dispersing to the lungs and other organs (Fig 4). In antiretroviral-naïve humans with latent TB co-infected with HIV, abnormal FDG uptake in lymph nodes was associated with reactivation. Ten participants determined to have subclinical TB pathology were more likely to develop abnormal uptake of FDG in thoracic lymph nodes compared to participants without subclinical TB disease. Participants with subclinical TB pathology were also significantly more likely to develop active TB disease (4/10) during the 6-month follow-up period compared with the 25 participants with no subclinical pathology of which none developed active TB disease [81]. These data suggest that reactivation of latent TB—whether by SIV/HIV infection, CD4 T-cell depletion, or TNF neutralization—can start in the lymph nodes and can be predicted by visualizing the metabolic activity of lymph nodes by PET-CT.

### Lymph nodes influence effectiveness of BCG vaccine

BCG is a live attenuated *M*. *bovis* strain and the only licensed vaccine for TB. It is effective at protecting infants and children against the more serious forms of the disease such as miliary disease or TB meningitis but variable in efficacy in protecting against pulmonary TB in adults [82]. For a vaccine to successfully elicit an immune response, it is required to reach secondary lymphoid organs such as the lymph nodes. A study in C57BL/6 mice compared the efficacy of 3 BCG vaccination routes (intradermal [ID], subcutaneous [s.c.], and intralymphatic injection) in eliciting a robust immune response and protection from Mtb challenge [83]. Direct injection of BCG to the inguinal lymph nodes resulted in tremendous transient swelling of not just the injected lymph nodes but all the other lymph nodes as well (e.g., mesenteric, axillary, brachial, thoracic, and cervical nodes). This is in contrast to s.c. and ID vaccination, which caused minimal swelling of any of the lymph nodes examined. Lymph nodes from intralymphatically vaccinated mice harbored greater numbers of BCG by Ziehl-Neelsen staining compared to s.c.-vaccinated animals. Intralymphatic vaccination also elicited a more robust immune response compared to s.c.-vaccinated animals. Significantly more proliferation and stronger TNF, IL-2, IL-17, and IFNγ responses up to 40 days post vaccination were observed in purified protein derivative (PPD)-stimulated splenocytes from intralymphatic-vaccinated animals compared to s.c.-vaccinated animals. Direct vaccination of lymph nodes also resulted in significantly reduced Mtb burden (up to 12 weeks post infection) against Mtb challenge in the lungs and spleen compared to s.c. and unvaccinated control mice [83]. These data suggest that direct vaccination of lymph nodes could improve the efficacy of BCG in eliciting an immune response and protection against Mtb challenge.

Since lymph nodes are sites of Mtb infection and persistence [8], it is worth considering protection against Mtb infection in lymph nodes in preclinical vaccine studies. Two vaccination strategies have shown protection in both lungs and lymph nodes in macaques after Mtb infection. Vaccination with attenuated Mtb [84] and a cytomegalovirus vector encoding Mtb-specific antigens [85] in rhesus macaques have shown that macaques protected against Mtb challenge (e.g., mild disease and significantly lower Mtb burden in lungs) also showed an approximately 100-fold decrease in culturable Mtb in lung-draining lymph nodes. Complete protection against Mtb infection (i.e., no lung granuloma formation or formation of sterile granulomas) was best achieved in the context of Mtb reinfection in cynomolgus macaques [86] and intravenous (IV) BCG vaccination in rhesus macaques [87]. Using DNA barcoded Mtb libraries, cynomolgus macaques were infected with Mtb library A and after 16 weeks, rechallenged with Mtb library B. Macaques initially infected with Mtb library A developed significantly fewer library B lung granulomas, most of them sterile, compared to macaques only infected with Mtb library B (naïve controls). Importantly, Mtb library B only disseminated to the lymph nodes in 1 of 8 Mtb library A-infected macaques, and this was only in 1 lymph node. In contrast, 5 out of 6 naïve controls had one or more CFU+ lymph nodes [86]. IV BCG vaccination protected 6 out of 10 rhesus macaques from forming lung granulomas after Mtb challenge. Three of the remaining IV BCG vaccinated macaques were protected and formed ≤3 granulomas and had significantly lower lung CFU compared to the standard ID vaccination route. Overall, IV-BCG macaques had a 100,000-fold reduction in thoracic bacterial burden compared to the ID BCG group. Similar to the Mtb reinfection study, 9 out of 10 rhesus macaques in the IV-BCG vaccinated group did not grow Mtb in any of their lymph nodes examined. Based on these studies, a successful vaccine should induce rapid killing of Mtb when it enters the lungs and prevent Mtb from reaching the lymph nodes. When macaques were analyzed only 4 weeks after BCG vaccination (without Mtb challenge), only IV-BCG vaccinated animals had BCG in BAL, spleen, lung lobes, and peripheral and thoracic lymph nodes, while ID-vaccinated animals only harbored BCG in skin and draining axillary lymph nodes. Aerosol-vaccinated animals had BCG in lung lobes and BAL. At 2–4 weeks post-IV BCG vaccination, increased inflammation (FDG activity) in lung-draining lymph nodes, lung lobes, and spleen was observed by PET-CT; this was not seen in the other routes. IV-BCG vaccinated animals also had transient enlargement of the spleen and enlarged lymph nodes that contained non-necrotizing granulomas and increased proliferation in the B cell region, often with active germinal centers [87]. It seems that BCG infiltration and metabolic activity (probably activation of immune response) in thoracic lymph nodes and spleen sets IV BCG vaccinated macaques apart from other vaccination routes and may be important contributors to the astounding protection that IV BCG vaccination conferred against Mtb challenge. In both mice and NHP studies, BCG colonization of lung-draining lymph nodes is correlated with protection against Mtb challenge [83, 87]. In addition, prevention of Mtb infection in lymph nodes and not just the lungs should also be targeted when designing a vaccine. The role of lymph nodes in vaccine efficacy is worthy of additional study.

### Lower drug penetration in lymph nodes

Lymph nodes are sites where Mtb can persist, disseminate, and reactivate [51, 78–81]. Therefore, it is imperative that anti-TB drugs be tested for their ability to eliminate Mtb bacteria in the lymph nodes. Short-course drug treatment studies in cynomolgus macaques show that reduction in Mtb burden in lymph nodes is significantly impaired compared to lung granulomas (55-fold reduction in lymph nodes versus 181-fold reduction in lung granulomas [8]) in drug-treated versus untreated controls. Thus, anti-TB drugs are more effective in killing Mtb in lung granulomas compared to lymph nodes [8, 88]. There is only one study that the authors are aware of that examined concentrations of rifampicin (RIF) and isoniazid (INH), both first-line anti-TB drugs, in the blood, lungs, granulomas, and lymph nodes in humans [89, 90]. RIF had the highest concentration in the blood (6.95 μg/ml) followed by tuberculous foci (2.43 μg/g) and healthy lung tissue (2.22 μg/g). Thoracic lymph nodes (1.41 μg/g) had lower RIF concentration compared to blood and lung granulomas. Interestingly, the lowest RIF concentration was found in caseous lymph nodes (0.03 μg/g). In contrast, although INH concentration was also highest in the blood (4.11 μg/ml), its concentration in healthy lungs (0.58 μg/g), bronchopulmonary lymph nodes (0.53 μg/g), cavities (0.59 μg/g), tuberculous foci (0.6 μg/g), and caseous lymph nodes (0.21 μg/g) were all relatively similar [89, 90]. Remarkably, caseous lymph nodes had once again the lowest INH concentration. No information about Mtb burden in the different tissues was provided. Although this is just one study, it provides a glimpse of lower RIF and INH penetration in lymph nodes compared to lung granulomas. This lower drug penetration in lymph nodes could explain the reduced efficacy of anti-TB drugs in killing Mtb in the lymph node compartment.

HIV also uses lymph nodes as latent reservoirs. Similar to the findings above, studies of drug penetration in lymph nodes in HIV patients show that concentrations of antiretroviral drugs in lymph nodes are significantly lower compared to the blood [91–93]. By sequencing HIV DNA and RNA from blood and inguinal lymph nodes of HIV-infected patients at different time intervals post antiretroviral treatment, Lorenzo-Redondo and colleagues discovered that despite undetectable levels of viral RNA in plasma during treatment, low-level viral replication still occurs in lymph nodes and this phenomenon was attributed to low antiretroviral drug penetration in these organs [91, 92]. Similar findings were reported in a study of 12 HIV-infected patients after antiretroviral drug initiation. Fletcher and colleagues showed that the antiretroviral drug concentrations in lymph nodes were significantly lower compared to the blood, and this correlated with continuous HIV replication [93]. Thus, lower drug penetration in lymph nodes is not unique to TB.

## Concluding remarks

Lymph nodes are underappreciated in the study of TB. It is clear that aside from their main function of initiating and shaping adaptive immune responses, lymph nodes also serve as niches for Mtb growth and persistence. Although lymph nodes mount an immune response to Mtb infection, data support that they are poor killers of Mtb [8]. In addition, latent TB reactivation can originate from lymph nodes [51, 78–81]. As such, eliminating Mtb in these organs requires closer attention. Current anti-TB drug regimens are less efficient in reducing Mtb burden in the lymph nodes compared to lung granulomas, which may be attributed to poor penetration of drugs in the involved lymph nodes. Vaccine and anti-TB drug trials should examine efficacy of preventing Mtb infection or eliminating Mtb in lymph nodes and not just the lungs. Tools (e.g., nanomaterials) and efficient antigen or drug design can also be used to target anti-TB drugs and vaccines to the lymph nodes. Further studies in improving vaccine and drug delivery to the lymph nodes is warranted.

## Methods

### Mtb dissemination from lungs to lymph nodes analysis

Cynomolgus macaques (*Macaca fascicularis*) (*n* = 74) that served as controls (no vaccine or drug treatment) for other studies from 2011 to 2018 were selected for this study. These macaques were infected with 2–92 CFU (median = 8 CFU) of Mtb Erdman using a bronchoscope. The animals used in this study are summarized in S1 Table. Existing records were reviewed for the location of granulomas in the lungs and CFU positivity of lymph nodes in the same animal. Data were graphed using GraphPad Prism version 8 (GraphPad Software, San Diego, CA). All procedures and protocols were approved by the University of Pittsburgh’s Institutional Animal Care and Use Committee (IACUC).

### Histology

Histological examination was performed by an experienced veterinary pathologist (E. Klein) as previously described [39]. Lymph nodes obtained during necropsy were cut (4–6 mm) and stained with hematoxylin–eosin. Characteristics of granulomas, such as size, type (caseous, non-necrotizing, suppurative, or mixed), distribution pattern (focal, multifocal, coalescing, focally extensive, and locally invasive), and cellular composition were noted. The list of animals used in this review are summarized in S2 Table.

### Ethics statement

All experimental manipulations, protocols, and care of the animals were approved by the University of Pittsburgh School of Medicine IACUC. The protocol assurance number for our IACUC is A3187-01. Our specific protocol approval numbers for this project are as follows: 1105870, 11090030, 11110045, 12060181, 12080653, 12090832, 13122856, 14023305, 14043492, 15055811, 15066174, 15126588, 16017370, 17029987, and 17060529. The IACUC adheres to national guidelines established in the Animal Welfare Act (7 U.S.C. Sections 2131–2159) and the Guide for the Care and Use of Laboratory Animals (8th Edition) as mandated by the US Public Health Service Policy.

All macaques used in this study were housed at the University of Pittsburgh in rooms with autonomously controlled temperature, humidity, and lighting. Animals were singly housed in caging at least 2 m^2^ apart that allowed visual and tactile contact with neighboring conspecifics. The macaques were fed twice daily with biscuits formulated for NHPs, supplemented at least 4 days/wk with large pieces of fresh fruits or vegetables. Animals had access to water ad libitum. Because our macaques were singly housed due to the infectious nature of these studies, an enhanced enrichment plan was designed and overseen by our NHP enrichment specialist. This plan has 3 components. First, species-specific behaviors are encouraged. All animals have access to toys and other manipulata, some of which will be filled with food treats (e.g., frozen fruit, peanut butter, etc.). These are rotated on a regular basis. Puzzle feeders, foraging boards, and cardboard tubes containing small food items also are placed in the cage to stimulate foraging behaviors. Adjustable mirrors accessible to the animals stimulate interaction between animals. Second, routine interaction between humans and macaques are encouraged. These interactions occur daily and consist mainly of small food objects offered as enrichment and adhere to established safety protocols. Animal caretakers are encouraged to interact with the animals (by talking or with facial expressions) while performing tasks in the housing area. Routine procedures (e.g., feeding, cage cleaning, etc.) are done on a strict schedule to allow the animals to acclimate to a routine daily schedule. Third, all macaques are provided with a variety of visual and auditory stimulation. Housing areas contain either radios or TV/video equipment that play cartoons or other formats designed for children for at least 3 hours each day. The videos and radios are rotated between animal rooms so that the same enrichment is not played repetitively for the same group of animals.

All animals are checked at least twice daily to assess appetite, attitude, activity level, hydration status, etc. Following Mtb infection, the animals are monitored closely for evidence of disease (e.g., anorexia, weight loss, tachypnea, dyspnea, coughing). Physical exams, including weights, are performed on a regular basis. Animals are sedated prior to all veterinary procedures (e.g., blood draws, etc.) using ketamine or other approved drugs. Regular PET-CT imaging is conducted on most of our macaques following infection and has proved very useful for monitoring disease progression. Our veterinary technicians monitor animals especially closely for any signs of pain or distress. If any are noted, appropriate supportive care (e.g., dietary supplementation, rehydration) and clinical treatments (analgesics) are given. Any animal considered to have advanced disease or intractable pain or distress from any cause is sedated with ketamine and then humanely euthanatized using sodium pentobarbital.

## Supporting information

S1 Table List of animals used in studying Mtb dissemination from lungs to lymph nodes. Mtb, *Mycobacterium tuberculosis*
(DOCX)

S2 Table List of animals used for histology. (DOCX)
