# Supplementary material for: Lymph nodes—The neglected battlefield in tuberculosis
Source: PLoS Pathog. 2020 Aug 13;16(8):e1008632. doi: 10.1371/journal.ppat.1008632 (PMC7425845; doi:10.1371/journal.ppat.1008632)
Supplement: S1 Table — Mtb, Mycobacterium tuberculosis (DOCX) [file ppat.1008632.s001.docx]

| Monkey ID | Days post-infection | Weeks post-infection | Lung disease side | CFU+ LN side |
| --- | --- | --- | --- | --- |
| 13516 | 90 | 13 | Bilateral | Bilateral |
| 30516 | 68 | 10 | Bilateral | Bilateral |
| 4017 | 69 | 10 | Bilateral | Bilateral |
| 21817 | 69 | 10 | Bilateral | Bilateral |
| 20212 | 72 | 10 | Bilateral | Bilateral |
| 20612 | 72 | 10 | Bilateral | Bilateral |
| 20912 | 72 | 10 | Bilateral | Bilateral |
| 15316 | 73 | 10 | Bilateral | Right |
| 2116 | 74 | 11 | Bilateral | Bilateral |
| 1114 | 81 | 12 | Bilateral | Right |
| 1314 | 81 | 12 | Bilateral | Bilateral |
| 17413 | 83 | 12 | Bilateral | Bilateral |
| 9515 | 83 | 12 | Bilateral | Bilateral |
| 20215 | 83 | 12 | Bilateral | Bilateral |
| 18214 | 84 | 12 | Bilateral | Bilateral |
| 17111 | 84 | 12 | Bilateral | Bilateral |
| 2512 | 85 | 12 | Bilateral | Bilateral |
| 20415 | 85 | 12 | Bilateral | Left |
| 13716 | 88 | 13 | Bilateral | Bilateral |
| 13616 | 89 | 13 | Bilateral | Left |
| 18314 | 91 | 13 | Bilateral | Bilateral |
| 9611 | 92 | 13 | Bilateral | Bilateral |
| 5816 | 104 | 15 | Bilateral | Bilateral |
| 18514 | 118 | 17 | Bilateral | Bilateral |
| 17013 | 119 | 17 | Bilateral | Bilateral |
| 18414 | 125 | 18 | Bilateral | Bilateral |
| 17713 | 129 | 18 | Bilateral | Left |
| 21112 | 133 | 19 | Bilateral | Bilateral |
| 20512 | 143 | 20 | Bilateral | Right |
| 20712 | 152 | 22 | Bilateral | Bilateral |
| 4316 | 160 | 23 | Bilateral | Bilateral |
| 4216 | 167 | 24 | Bilateral | Right |
| 16013 | 170 | 24 | Bilateral | Bilateral |
| 31016 | 175 | 25 | Bilateral | Left |
| 3916 | 188 | 27 | Bilateral | Bilateral |
| 9511 | 198 | 28 | Bilateral | Bilateral |
| 9815 | 242 | 35 | Bilateral | Bilateral |
| 3817 | 68 | 10 | Left | Left |
| 20315 | 83 | 12 | Left | Left |
| 1714 | 91 | 13 | Left | Left |
| 15416 | 102 | 15 | Left | Left |
| 2016 | 109 | 16 | Left | Left |
| 23018 | 117 | 17 | Left | Bilateral |
| 9711 | 175 | 25 | Left | Left |
| 21517 | 183 | 26 | Left | Left |
| 30916 | 226 | 32 | Left | Left |
| 9811 | 238 | 34 | Left | Bilateral |
| 2312 | 330 | 47 | Left | Left |
| 2712 | 384 | 55 | Left | None |
| 4217 | 71 | 10 | Right | Right |
| 3917 | 72 | 10 | Right | Bilateral |
| 21917 | 74 | 11 | Right | Right |
| 21217 | 75 | 11 | Right | Right |
| 28816 | 75 | 11 | Right | Right |
| 22017 | 77 | 11 | Right | Right |
| 9714 | 78 | 11 | Right | Right |
| 9814 | 78 | 11 | Right | Bilateral |
| 20115 | 81 | 12 | Right | Right |
| 2412 | 83 | 12 | Right | Bilateral |
| 9415 | 85 | 12 | Right | Bilateral |
| 616 | 88 | 13 | Right | Right |
| 916 | 95 | 14 | Right | Right |
| 5217 | 112 | 16 | Right | None |
| 4317 | 119 | 17 | Right | Right |
| 16213 | 163 | 23 | Right | Right |
| 16113 | 170 | 24 | Right | Bilateral |
| 20715 | 174 | 25 | Right | Right |
| 20915 | 202 | 29 | Right | Right |
| 2916 | 207 | 30 | Right | Right |
| 4116 | 217 | 31 | Right | None |
| 14112 | 284 | 41 | Right | Right |
| 15712 | 293 | 42 | Right | Right |
| 17211 | 328 | 47 | Right | Right |
| 2612 | 379 | 54 | Right | Right |
