# Supplementary material for: Lymph nodes—The neglected battlefield in tuberculosis
Source: PLoS Pathog. 2020 Aug 13;16(8):e1008632. doi: 10.1371/journal.ppat.1008632 (PMC7425845; doi:10.1371/journal.ppat.1008632)
Supplement: S2 Table — (DOCX) [file ppat.1008632.s002.docx]

| Monkey ID | Species | Days post-infection | Weeks post-infection |
| --- | --- | --- | --- |
| 20512 | Cynomolgus | 143 | 20 |
| 16013 | Cynomolgus | 170 | 24 |
| 8214 | Rhesus | 186 | 27 |
| 7114 | Rhesus | 195 | 28 |
